# Polyethylene glycol vs. sodium phosphate for bowel preparation: A treatment arm meta-analysis of randomized controlled trials

**DOI:** 10.1186/1471-230X-11-38

**Published:** 2011-04-14

**Authors:** Ravi Juluri, George Eckert, Thomas F Imperiale

**Affiliations:** 1 Indiana University Health Physicians, Indianapolis, Indiana, USA; 2 Department of Medicine, Division of Biostatistics, Indiana University School of Medicine, Indianapolis, Indiana, USA; 3 Department of Medicine, Division of Gastroenterology and Hepatology, Indiana University School of Medicine, Indianapolis, Indiana, USA; 4 Regenstrief Institute, Inc, Indianapolis, Indiana, USA; 5 Center of Excellence for Implementing Evidence-based Practice, Richard L. Roudebush VAMC, Indianapolis, Indiana, USA

**Keywords:** Colonoscopy, Bowel preparation, Polyethylene Glycol, Sodium Phosphate

## Abstract

**Background:**

Results of meta-analyses of randomized trials comparing PEG and NaP are inconsistent and have not included trials comparing either or both preps to less traditional ones.

**AIM:** To perform a meta-analysis by treatment arm.

**Methods:**

Using MEDLINE and EMBASE, we identified English-language trials published from 1990 to 2008 that included PEG and/or NaP, and aggregated them by treatment arm into: 4 liter (L) PEG; 2 L PEG; split-dose PEG; two 45 ml doses of NaP +/- adjunctive medication; and NaP tablets. We compared prep quality and the proportion completing the prep.

**Results:**

Among 71 trials (patient N = 10,201), excellent prep quality was present in 34% (CI, 26-41%) for 4 L PEG alone; 39% (CI, 26-51%) for 2 L PEG; 37% (CI, 28-46%) for split-dose PEG; 42% (CI, 33-51%) for NaP solution; 44% (CI, 38-51%) for NaP with adjunctive meds; and 58% (CI, 49-67%) for NaP tablets. Patients receiving NaP were more likely to complete the prep (97% [CI, 96-98%] vs. 90% [CI, 87-92%] for 4L PEG alone); however, completion rates for 2L PEG (98%) and split dose PEG (95%) were similar to NaP.

**Conclusions:**

NaP tablets resulted in better prep quality and higher completion rates compared to other regimens. In comparisons limited by sample size, split dose PEG was not statistically different from NaP solution for completion rate or prep quality.

## Background

Colonoscopy is a well-established procedure for screening, diagnosis and treatment of colorectal disorders [1,2]. For colonoscopy to be effective, adequate preparation of the bowel is required for visualization of the colonic mucosa. To achieve this, a bowel preparation should be tolerable, safe, effective and convenient. Bowel preparation is considered to be the main obstacle for patients undergoing colonoscopy [3]. The aversion toward bowel preparation may be related to its taste, fluid volume ingested, or side effects such as nausea, bloating and vomiting.

Polyethylene glycol (PEG) (NuLYTELY, Half Lytely, and GoLYTELY; Braintree Laboratories, Inc, Braintree, MA; Colyte; Schwarz Pharma, Milwaukee, WI, and MoviPrep; Salix Pharmaceutical, Inc, Morrisville, NC) and sodium phosphate (NaP) tablets (Visicol and OsmoPrep Tablets; Salix Pharmaceuticals, Inc, Morrisville, NC), NaP solution (Fleet Phospho-soda; C.B. Fleet Company, Inc, Lynchburg, VA), are the most widely used agents for colon cleansing. Polyethylene glycol is an orally administered isotonic solution introduced in 1980 [4]. Since PEG is nondigestible and nonabsorbable, it cleanses the colon by purging of intraluminal contents [5]. Because it is iso-osmolar with plasma, the large volume of PEG does not result in significant fluid shifts. It has been shown to be highly effective when taken as instructed (4L of PEG solution). However, the efficacy of standard 4 L PEG outside of clinical trials is compromised by poor patient compliance. The large volume and taste are the main factors that contribute to poor patient compliance and tolerability [6-8], which led to development of reduced volume PEG solution with or without laxatives, sulfate-free, and flavored PEG solutions (Half Lytely, NuLYTELY) in an attempt to reduce the sulfate odor and improve taste [9]. In some studies, split-dose PEG has been more effective than standard 4L PEG [10,11].

Sodium phosphate (NaP), a buffered saline laxative, gained popularity as an alternative method for colonic preparation due to its smaller volume. Containing monobasic sodium phosphate and dibasic sodium phosphate, NaP acts as an osmotic laxative, cleansing the colon by drawing fluids into the gastrointestinal tract. Several randomized trials and meta-analyses comparing PEG and NaP have suggested that NaP is safe, better tolerated, cost-effective, and equally or more effective [5,12-15]. NaP tablets (Visicol ^®^) were designed to improve the taste and reduce the volume required for bowel preparation. NaP tablets contain microcrystalline cellulose which can be deposited in the colon requiring additional irrigation. A newer residue-free formulation of sodium phosphate tablets (OsmoPrepTM) was introduced [16] to overcome this limitation.

Previous meta-analyses, [5,17,18] have included head-to-head trials of PEG vs. NaP but have not included trials comparing either or both of these preps to other, less commonly used preps. The objective of this meta-analysis was to quantify and compare the effect of the two bowel preps on efficacy of and patient adherence to NaP vs. PEG for elective colonoscopy.

## Methods

### Search Strategy and selection criteria

We searched the medical literature from January 1990 to December 2008 using MEDLINE and EMBASE bibliographic databases and identified all relevant English language publications. The search strategy used the following MeSH terms: 1) colonoscopy, 2) polyethylene glycol, 3) phosphates, 4) cathartics and 5) bowel prep. We limited these sets of articles to diagnostic and therapeutic uses and to human studies published in English. In addition, we hand-searched the reference lists of every selected primary study for additional trials. The following criteria were used to select studies for inclusion: 1) study design: randomized controlled trial (RCT), 2) patient population: adult patients undergoing elective colonoscopy, 3) year of publication (1990-2008), 4) dosing and frequency schedules of PEG and NaP commonly used in clinical practice. We excluded duplicate trials, those that lacked categorical data on both prep quality and adherence; review articles; editorials; and letters to the editor.

### Assembling the treatment arms

The analysis compared on treatment arms rather than individual trials. Each trial was disassembled and aggregated by treatment arm [19,20] into one of the following groups: 1) 4 liter PEG +/- adjunctive medications (e.g., dulcolax), 2) 2 liter PEG, 3) Split-dose PEG, 4) NaP solution - two 45 ml doses +/- adjunctive medications, 5) NaP tablets. Disassembly of the trials into treatment arms was based on the determination that the treatment arms were clinically homogeneous in composition. This determination was based on a qualitative assessment of similarity of the trial populations, study settings, prep regimens, ratings of bowel prep quality, and outcomes. All descriptive and quantitative data were extracted from the papers to an analytic database. If the data for the particular variable were not available, that variable was excluded from analysis and no assumption was made about the missing data.

### Quantitative analysis

Descriptive data were extracted to determine clinical similarity of the individual trials; extracted quantitative data included the number of subjects in each treatment arms and those with each outcome. Discrepancies in data extraction were resolved in discussion. For pooling procedures, the extracted data were combined across treatment arms rather than across individual trials. We assumed the presence of clinical heterogeneity because of variation in factors that were not consistently described in each trial, such as prep timing, consumption of additional liquids, and dietary instructions. We combined the data using the random effects model developed by DerSimonian-Laird [21], which adjusts for variation within treatment arms and provides a more conservative estimate an effect by providing wider confidence intervals (CIs). We compared prep quality (excellent, good, fair, poor) and the percent of persons completing the prep using weighted, summary- level proportions and 95% CI. All analyses and calculations were done using r-meta library (version 2.14) for the statistical software R (version 2.5.1).

## Results

### Descriptive findings

**Figure 1:**
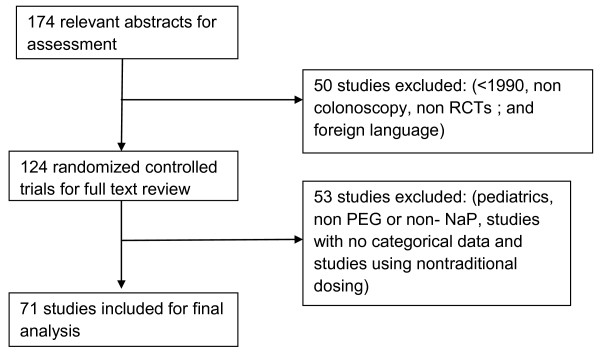
**Flow chart diagram for the studies identified in the meta-analysis**.

One hundred seventy four abstracts were obtained from 1990 through 2008 using MEDLINE and EMBASE; 50 were excluded as they were either published prior to 1990 (n = 18), involved bowel preparation for non colonoscopy use (n = 11), were published in foreign language (n = 8), or were non-randomized controlled trials (n = 13). Of the 124 randomized controlled trials included for full text review, 53 trials were excluded. The number of articles and reasons for exclusion were as follows: trials which included a pediatric population (n = 6); trials that did not include PEG or NaP (n = 24); trials with no categorical data (n = 12); and trials with non-traditional doses of either prep (e.g., single dose 3L or 6L PEG solutions regimen, single dose NaP (45 mL or 90 mL; n = 11) were excluded (Figure 1).

For analysis, we included 71 randomized controlled trials [6-8,10-14,16,22-83] involving 10,201 patients. Trial aggregation by treatment arm resulted in the following prep arms: 4 liter PEG with and without adjunctive medications (e.g., metoclopramide, dulcolax); 2 liter PEG; split-dose PEG; NaP solution - two 45 ml doses with and without adjunctive medications; and NaP tablets. All low volume PEG trials (i.e., 2 liter) invariably used an adjunctive medication such as bisacodyl (70%), senna (20%), and magnesium citrate or ascorbic acid (10%). Trials that used split dose PEG regimen either divided a 4 liter dose into 2 liter the day before and 2 liter on the day of the procedure or divided a 3 liter dose into 2 liter the day before and 1 liter the day of the procedure.

Descriptive data for each treatment arm is shown in Table 1. Overall mean age was 58 years; 49% of the study participants were men. At least 68 (95.7%) of 71 trials were investigator-blinded. The trials had comparable study populations; individual trial inclusion criteria consisted of patients with indications for screening or diagnostic colonoscopy. Exclusion criteria generally included congestive heart failure, recent myocardial infarction, renal insufficiency, and cirrhosis with ascites. All trials used comparable scales for rating bowel prep quality [6]: excellent prep quality was defined as a small volume of clear liquid or greater than 95% of surface seen; good prep quality was defined as large volume of clear liquid covering 5% to 25% of the surface but greater than 90% of surface seen; fair prep quality was defined as some semi-solid stool that could be suctioned or washed away but greater than 90% of surface seen; and poor prep quality was defined as: semi-solid stool that could not be suctioned or washed away and less than 90% of the surface seen. A few trials defined prep quality as "excellent or good", satisfactory (excellent or good), or unsatisfactory (fair or poor). These definitions were comparable to the individual quality components of the four point scale for prep quality [6].

**Table 1:** Descriptive data for each prep treatment arm

Prep Treatment Arm	# of Treatment Arms	# of Pts	Mean Age, y (range)	% Males (range)
NaP Solution alone	31	2463	58.2 (51-84)	46.6 (20-77)
NaP Solution with adjunctive meds	5	358	56.3 (52-60)	44.6 (31-52)
NaP Tablets	7	1793	56.3 (56-58)	43.7 (40-49)
4L PEG alone	33	2729	59.3 (52-84)	48.2 (14-99)
4L PEG with adjunctive meds	6	372	63.3 (58-81)	57.9 (34-100)
2L PEG	17	1919	58.6 (54-62)	52.6 (30-99)
Split dose PEG	5	567	55.3 (52-57)	49.6 (37-65)

The method of preparation of PEG and NaP were similar among the trials with some variation in the timing of prep consumption. Dietary recommendations the day before colonoscopy varied from regular to a clear liquid diet for lunch to a full clear liquid diet in the evening. Co-interventions accompanying trials with 2 liter PEG, 4 liter PEG with adjunctive medications, and NaP solution with adjunctive medications were either taken separately and only rarely in combination with the study preparation, and included magnesium citrate, metoclopramide, psyllium, bisacodyl, cisapride, ascorbic acid, senna, and simethicone.

### Quantitative findings

The proportion of persons with excellent prep quality were 42.1% (CI, 33-51%) for NaP solution alone; 44.4% (CI, 38-51%) for NaP with adjunctive meds; 58.2% (CI, 49-67%) for NaP tablets; 33.7% (CI, 26-41%) for 4 liter PEG alone; 38.7% (CI, 26-51%) for 2 literL PEG; and 37.2% (CI, 28-46%) for split-dose PEG (Table 2). Based on the criterion of minimal or no overlap of the 95% CIs, NaP tablets resulted in a greater likelihood of achieving an excellent quality prep than did all PEG groups, while NaP solution was intermediate. All PEG groups were essentially equivalent with respect to prep quality (Table 2).

**Table 2:** Prep quality by treatment arm

Prep Treatment Arm	# of Treatment Arms	% Excellent (95% CI)	% Good (95% CI)	% Fair (95% CI)	% Poor (95% CI)
NaP Solution alone	22	42.1 (33-51)	31.0 (25-37)	16.4 (13-20)	7.0 (5-9)
NaP Sol'n with adjunctive meds	3	44.4 (38-51)	23.0 (18-28)	26.2 (21-32)	5.6 (2-9)
NaP tablets	7	58.2 (49-67)	28.8 (22-36)	9.9 (7-14)	1.4 (0.6-2)
4L PEG alone	23	33.7 (26-41)	36.8 (33-41)	23.3 (15-31)	5.9 (4-8)
2L PEG	11	38.7 (26-51)	38.6 (29-48)	23.5 (13-34)	4.3 (2-6)
Split dose PEG	5	37.2 (28-46)	43.0 (35-51)	11.1 (5-17)	5.7 (1-10)

**Figure 2:**
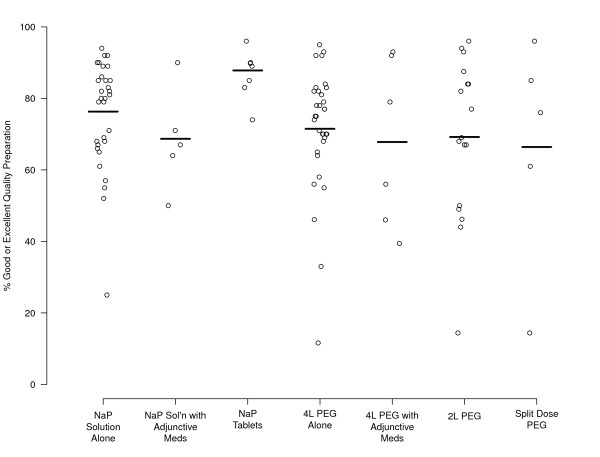
**Forest plot of preparation quality by treatment arm**.

The composite measure of excellent or good quality preparation was achieved by 76.3% (CI, 72-81%) of those who used NaP solution alone; by 68.7% (CI, 54-84%) of those who used NaP solution with adjunctive medication; and by 87.8% (CI, 83-93%) of those who used NaP tablets (Table 3 and Figure 2). For the PEG treatment subgroups, an excellent or good prep quality was achieved by 71.5% (CI, 64-80%) for 4 liter PEG alone; in 67.8% (CI, 49-87%) for 4 liter PEG with adjunctive medications; in 69.2% (CI, 58-81%) for 2 liter PEG; and in 66.4% (CI, 31-100%) for split-dose PEG. Use of NaP tablets was more likely to result in good or excellent quality prep than both NaP solution groups. When compared to the PEG groups, NaP tablets were superior to both 4 liter and 2 liter PEG groups and were superior to 4 liter PEG with adjunctive medications, with minimally overlapping CIs. All comparisons that included split-dose PEG resulted in significant overlap of the 95% CIs because of both the relatively small numbers of subjects in this group and the variation in results among the individual studies. Thus, while split-dose PEG was not statistically different from any of the other groups, the proportion of subjects with a good or excellent quality prep was numerically lower than all groups and to a clinically-important degree as compared with NaP tablets.

**Table 3:** Prep quality (Excellent/Good) and completion rates by treatment arm

Prep Treatment Arm	# of Treatment Arms	# of Pts	% Good or Excellent (95% CI)	% Prep Completed (95% CI)
NaP Solution alone	31	2463	76.3 (72-81)	97.3 (96-98)
NaP Sol'n with adjunctive meds	5	358	68.7 (54-84)	92.9 (85-99)
NaP Tablets	7	1793	87.8 (83-93)	97.2 (95-99)
4L PEG alone	33	2729	71.5 (64-80)	89.5 (87-92)
4L PEG with adjunctive meds	6	372	67.8 (49-87)	95.3 (86-100)
2L PEG	17	1919	69.2 (58-81)	98.0 (96-100)
Split dose PEG	5	567	66.4 (31-100)	95.4 (86-100)

**Figure 3:**
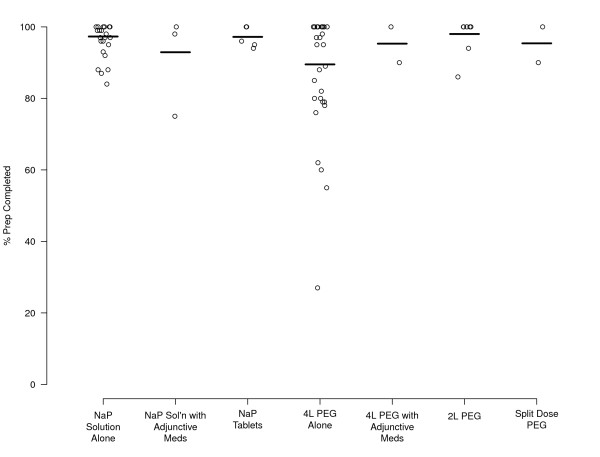
**Forest plot of preparation completion/acceptability**.

Prep completion rates are shown in Table 3 and Figure 3. Patients who received NaP either alone in liquid form or in tablet form were more likely to complete the prep (97.3% [CI, 96-98%] and 97.2% [CI, 95-99] respectively, vs. 89.5% [CI, 87-92%] for PEG). However, completion rates for 2L PEG (98%) and split-dose PEG (95%) were similar to NaP.

## Discussion

This meta-analysis examined 71 randomized controlled trials that included NaP or PEG solution or both for bowel preparation prior to elective, outpatient colonoscopy. The findings indicate that NaP resulted in an excellent quality prep more often than PEG. Further, based on minimal or no overlap of the 95% CIs, NaP tablets resulted in a greater likelihood of achieving an excellent quality prep than did all PEG groups, while NaP solution was in between. There was no difference in prep quality among the various PEG subgroups. Among treatments arms where prep quality could be quantified as a composite of excellent or good, NaP tablets (87.8%) were numerically superior to all other forms of either prep. There was minimal overlap of the 95% CI of NaP tablets with those of both NaP solution and 4 L PEG arms, both of which included adjunctive medications. Despite an absolute difference of just over 21% between NaP tablets and split dose 4 L PEG that favored NaP tablets, the CIs showed a large degree of overlap, most likely due to the imprecision of the individual trial point estimate for split dose PEG. Finally, among the trials that included completion rates, NaP was more likely to be completed than PEG, with the exception of the split dose PEG regimen.

Previous meta-analyses of head-to-head trials of PEG vs. NaP have reported that sodium phosphate is more effective, better tolerated, and less costly than PEG [5,18]. However, in 2007 a meta-analysis by Belsey *et al *reported that no single bowel preparation was consistently superior to others [17]. Both meta-analyses excluded data from trials comparing either or both of these preps to other, less commonly used preps.

This analysis has strengths and limitations. One strength is the breadth of our search strategy and analysis, as we included studies that have been excluded in other systematic reviews. Another strength is the clinical homogeneity of the patient population studied: all groups were comprised of outpatients undergoing elective colonoscopy. Further, as best we could determine, the study populations, even after re-assembly by treatment arm, appear to be demographically and clinically comparable. Finally, the large sample size of this analysis provides reasonable precision for most of the point estimates of effect for both efficacy and tolerability.

With regard to limitations, the potential for clinical heterogeneity is always present when combining trials, particularly for factors that were not measured. The possibility of clinical heterogeneity appears to be low; to minimize its effects, we used a random effects model, which accounts for heterogeneity by both providing a point estimate that is less weighted by the studies with larger sample sizes and resulting in wider confidence intervals. Several candidate factors may contribute to clinical and/or methodological heterogeneity among trials. One factor is variation in timing of bowel prep. The time at which the bowel prep was started was not uniform among the trials ranging from 2:00 PM in the afternoon to 7:00 PM in the evening the day before the scheduled procedure. This may have affected those patients undergoing colonoscopy in the afternoon by affecting prep quality particularly in the right colon, where intestinal chyme can accumulate, obscuring the mucosa. Another factor potentially contributing to heterogeneity is the variation in dietary instructions prior to and during the prep, which also were not uniform among the trials, and which ranged from a regular diet to a clear liquid diet for lunch and clear liquid diet in the evening.

A second limitation is the uncertain acceptability of the "treatment-arm" method of doing meta-analysis. While this method has been used previously for comparing treatments for rheumatoid arthritis [19], for prevention of deep venous thrombosis following total hip replacement [84] and for treatment of premature labor [85], its validity is less well established than is head-to-head meta-analysis where comparators are the same in all studies. Limiting the analysis to a head-to-head comparison would not have allowed consideration of evidence from trials where either NaP or PEG was compared to another bowel preparation. An alternative to our "treatment-arm" approach is a mixed treatment comparison or "network" meta-analysis, which is another way of quantitatively aggregating data across studies containing disparate comparators [86,87]. It allows comparison of multiple treatments, combining direct and indirect evidence in a single analysis. While head-to-head meta-analysis and network meta-analysis of the same data have been compared [88], there are no comparative analyses between network and treatment-arm meta-analyses. In the absence of such comparative data, it remains uncertain which method is most appropriate for synthesizing quantitative data, and under which circumstances the two methods differ in results.

A third potential factor is the variation in definitions of patient tolerance of the prep. While some trials defined patient tolerance by different parameters (e.g. completion rates, willingness to repeat the prep, palatability and adverse affects), others defined patient tolerance as a single parameter and reported it as a single cumulative estimate.

In recent years, three reports have described 22 patients who developed renal insufficiency due to nephrocalcinosis that was temporally associated with use of NaP for colonoscopy prep, 4 of whom progressed to end stage renal disease requiring dialysis [89-91]. The majority of these patients had co-morbid conditions such as diabetes mellitus, hypertension (treated with angiotensin-converting enzyme inhibitors [ACE-I] or angiotensin receptor blockers [ARBs] or diuretics), preexisting renal insufficiency, were elderly, or had small bowel disease that resulted in calcium and vitamin D malabsorption. Renal biopsies of many of the cases showed nephrocalcinosis with intra-tubular deposition of calcium-phosphate. The term for this pathologic condition is acute phosphate nephropathy (APN). The histopathology suggests that sodium phosphate ingestion leads to obstructive calcium-phosphate crystalluria followed by acute intra-tubular nephrocalcinosis. These reports have recently raised concerns that led Food and Drug Administration [92] to announce a safety alert in December 2008, stating that a boxed warning would be added to the labeling on prescription OSPs (Visicol and OsmoPrep) and recommending against use of over-the-counter OSPs for bowel preparation. Shortly after this announcement, all over-the-counter NaP products were voluntarily removed from the market, with a subsequent sharp decline in use of NaP solution.

Despite the FDA's action and resulting reaction, the published data suggest that absolute risk of APN is very low [93,94]. Further, a recent systematic review and meta-analysis of seven controlled studies (patient N = 14,520) of the effects of NaP versus comparator on kidney function showed that there is significant clinical heterogeneity in the populations studied, study methods, definition of kidney injury, and results [95]. Quantitatively, the pooled odds ratio for kidney injury among NaP-treated patients ranged from 1.08 (CI, 0.71-1.62) to 1.22 (CI, 0.77-1.92). The investigators concluded that it was not possible to discern whether there is a true association between NaP and kidney injury.

The results of this meta-analysis apply to patients undergoing elective colonoscopy who do not have a history of co-morbid conditions such as renal insufficiency, recent myocardial infarction, ascites due to cirrhosis, and congestive heart failure. Further, NaP should not be used by patients with suspected or established inflammatory bowel diseases because of aphthous ulcerations it may cause [96]. While NaP solution is not currently available, tablet forms of NaP remain available by prescription only. Physicians should be aware of the risk of acute kidney injury with NaP preparations and should not use it in older patients, in those with preexisting renal insufficiency, and in patients on medications that can affect volume status or renal function (diuretics, ACE inhibitors, and ARBs). Further, all patients should be encouraged to adequately hydrate themselves prior to and while using NaP preparations.

## Conclusions

In conclusion, this treatment-arm meta-analysis of NaP and PEG suggests that NaP tablets result in a better quality prep than NaP solution, 2L PEG, and 4 L PEG alone. NaP is more likely to be completed overall. In comparisons limited by sample size, split dose PEG was not statistically different from NaP for both completion rate and prep quality. Head-to-head trials between split-dose PEG and NaP tablets would be useful in further defining the relative efficacy of these two regimens.

## Competing interests

The authors declare that they have no competing interests.

## Authors' contributions

**RJ**: carried out data collection, analysis and interpretation of the data, sequence alignment and drafting the manuscript. **GE**: carried out the statistical analysis and helped in drafting relevant statistical discussion in the manuscript. **TFI**: carried out- analysis and interpretation of the data, conceived of the analysis, participated in sequence alignment and final approval of the manuscript. All authors read and approved the final manuscript.

## Pre-publication history

The pre-publication history for this paper can be accessed here:

http://www.biomedcentral.com/1471-230X/11/38/prepub
